# Complete chloroplast genome sequence of *Populus pruinosa* Schrenk from PacBio Sequel II Platform

**DOI:** 10.1080/23802359.2020.1824593

**Published:** 2020-09-29

**Authors:** Zhong-Shuai Gai, Rui Liao, Zhen-Bo Jiang, Pei-Pei Jiao, Shan-He Zhang, Rui Qin, Hong Liu, Zhi-Hua Wu, Zhi-Jun Li

**Affiliations:** a Key Laboratory of Protection and Utilization of Biological Resources in Tarim Basin Xinjiang Production and Construction Corps, Tarim University, Alar, China; b Desert Poplar Research Center of Tarim University, Tarim University, Alar, China; c College of Life Science, Tarim University, Alar, China; d Hubei Provincial Key Laboratory for Protection and Application of Special Plant Germplasm in Wuling Area of China & Key Laboratory of State Ethnic Affairs Commission for Biological Technology, College of Life Sciences, South-Central University for Nationalities, Wuhan, China

**Keywords:** *Populus pruinosa*, chloroplast genome, PacBio Sequel, phylogeny

## Abstract

*Populus pruinosa* Schrenk plays an important role on ecological services in desert areas. The complete chloroplast genome was reported in this study using the PacBio Sequel II Platform. The chloroplast genome with a total size of 157,856 bp consists of two inverted repeats (IR, 27,673 bp) separated by a large single-copy region (LSC, 85,867 bp) and a small single-copy region (SSC, 16,645 bp). Further annotation revealed the chloroplast genome contains 111 genes, including 78 protein-coding genes, 29 tRNA genes, and 4 rRNA genes. A total of 151 simple sequence repeats (SSRs) were identified in the chloroplast genome. This information will be useful for study on the evolution and genetic diversity of *P. pruinosa* in the future.

*P. pruinose*, one of the few national protected plants, was mainly distributed along the bank of Tarim river, Yarkand river and Hotan rivers in Xinjiang, northwest of China. It usually formed a pure forest or a mixed forest with *P. euphratica* to maintain the balance of ecosystem, curb desertification and protect biodiversity in the extremely arid desert areas of Xinjiang (Zheng et al. 2016, 2019). In this study, to obtain the new insight into the phylogeny of *P. pruinose*, we sequenced, assembled, and annotated the accurate chloroplast genome with PacBio Sequel II platform.

The materials of *P. pruinosa* in this study were collected from *P. pruinosa* forest in the headwater region of the Tarim River on the northwestern margin of the Tarim basin in Xinjiang province of China (81°17'56.52″E, 40°32'36.90″N, 980 m above sea level). The voucher specimens of different ecotype (TD-00 TD-00194, TD-00302, TD-00304, TD-00306, TD-00310, and TD-00313, Populus pruinosa Schrenk) are stored in the herbarium of Tarim University, and the data related to the specimens are included in the database of wild plant germplasm resources of the Tarim basin (internal website, not yet open to the public; data available from the corresponding author upon reasonable request). The chloroplast DNA was extracted using a high-salt method and sequenced using the PacBio Sequel II platform. The whole chloroplast genome was assembled using Canu-1.7 (Koren et al. 2017) and 782 contigs with the N50 of 9552 bp were obtained. To assemble the complete chloroplast genome sequence, we aligned the contigs of a preliminary assembly to the whole chloroplast data from NCBI. Then, the draft genome was polished with Arrow (SMRT link-6.0.0, Pacific Biosciences, Menlo Park, CA). Due to the special structure of the chloroplast genome, we mapped the scaffolds to the reference to identify the two inverted repeats (IRs) and manually adjusted them. Then, the chloroplast gene structures were annotated using DOGMA (Wyman et al. 2004). The complete chloroplast genome was 157,856 bp (MT601900) and composed of two IRs of 27,673 bp each, which divide a large single copy (LSC) region of 85,867 bp and a small single copy (SSC) region of 16,645 bp, the average GC content was 36.54%. The chloroplast genomes encoded 111 functional genes, including 78 protein-coding genes, 29 tRNA genes, and 4 rRNA genes. A total of 151 SSR markers ranging from dinucleotide to hexanucleotide repeat motif were identified in *P. pruinose* chloroplast genome.

We aligned our assembled *P. pruinosa* chloroplast against that from Illumina (KX822074.1 in NCBI) using BLASTN, and found that the newly assembled genome got from PacBio platform was more accurate especially in the repeats. After designing the primer (5′-ACATCCAGTGCCAAAGTC-3′ and 5′-CCCTATTCCCTATTCTATTC-3′; 5′-GGGATGCTCCTAATAACC-3′ and 5′-ATTCTGTGGAAAGCCGTA-3′) for inconsistent positions between the genomes with two platforms, we verified the real existence of the insertion assembled by PacBio through Sanger sequencing. The result showed that the PacBio has the advantage of getting more complete chloroplast genome, which is also reported in other plants (Wu et al. 2014).

In our study, to explore the phylogenetic relationship of *P. pruinosa* within Salicaeae, additional 25 species from Salicaeae were studied. With the species of *Ricinus communis* as the outgroup, the phylogenetic trees were built from the whole protein-coding gene matrix by maximum-likelihood (ML) and Bayesian inference (BI) (Figure 1). The ML tree was generated using IQ-TREE (Nguyen et al. 2015) based on the best model of GTR + F+R2 and 1000 bootstrap replicates, and BI analysis was performed in MrBayes-3.2.6. This result showed that the analyzed *P. pruinosa* were closer to the species of *P. ilicifolia*.

**Figure 1.**
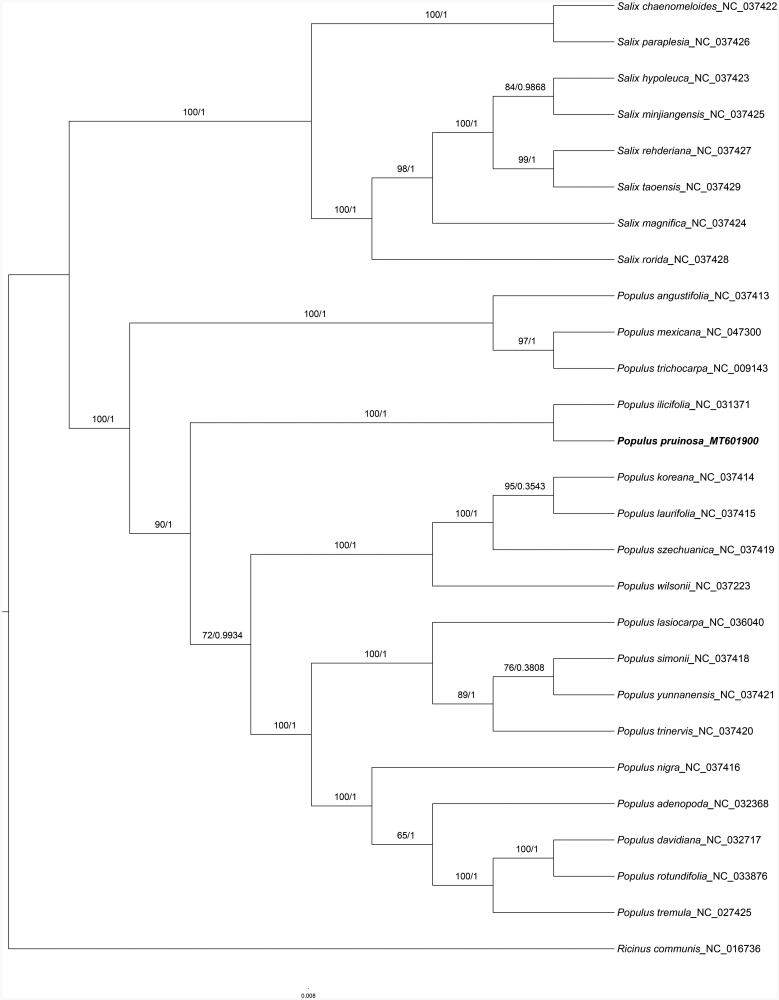
Phylogenetic tree inferred by both maximum-likelihood and Bayesian analysis from 27 species. The values on each node represent the bootstrap values from maximum-likelihood (left) and the posterior probability from Bayesian inference (right), respectively. The bold part is P. pruinosa in this study..

## Data Availability

The data that support the findings of this study are openly available in Genbank (https://www.ncbi.nlm.nih.gov/genbank/) according to their Genbank accession.

## References

[CIT0001] Koren S, Walenz BP, Berlin K, Miller JR, Bergman NH, Phillippy AM. 2017. Canu: scalable and accurate long-read assembly via adaptive k-mer weighting and repeat separation. Genome Res. 27(5):722–736. 28298431 10.1101/gr.215087.116 PMC5411767

[CIT0002] Nguyen L-T, Schmidt HA, von Haeseler A, Minh BQ. 2015. IQ-TREE: a fast and effective stochastic algorithm for estimating maximum-likelihood phylogenies. Mol Biol Evol. 32(1):268–274. 25371430 10.1093/molbev/msu300 PMC4271533

[CIT0003] Wu Z, Gui S, Quan Z, Pan L, Wang S, Ke W, Liang D, Ding Y. 2014. A precise chloroplast genome of *Nelumbo nucifera* (Nelumbonaceae) evaluated with Sanger, Illumina MiSeq, and PacBio RS II sequencing platforms: insight into the plastid evolution of basal eudicots. BMC Plant Biol. 14:289. 25407166 10.1186/s12870-014-0289-0 PMC4245832

[CIT0004] Wyman SK, Jansen RK, Boore JL. 2004. Automatic annotation of organellar genomes with DOGMA. Bioinformatics. 20(17):3252–3255. 15180927 10.1093/bioinformatics/bth352

[CIT0005] Zheng Y, Jiao P, Zhao Z, Li Z. 2016. Clonal growth of *Populus Pruinosa* Schrenk and its role in the regeneration of riparian forests. Ecol Eng. 94:380–392.

[CIT0006] Zheng Y, Zhai J, Chen J, Han Z, Jiao P, Li Z. 2019. Seasonal variations of clonal propagation characteristics of *Populus pruinosa* Schrenk, organ nutrient and soil fertility, and their coupling associations in the forest and forest edges. Bull Bot Res. 39(3):347–357.

